# Early Intervention Services for First Episode of Psychosis in South London and the Maudsley (SLaM): 20 Years of Care and Research for Young People

**DOI:** 10.3389/fpsyt.2020.577110

**Published:** 2020-11-24

**Authors:** Paolo Fusar-Poli, Serena Lai, Marta Di Forti, Eduardo Iacoponi, Graham Thornicroft, Philip McGuire, Sameer Jauhar

**Affiliations:** ^1^Early Psychosis: Interventions and Clinical-detection (EPIC) Lab, Department of Psychosis Studies, Institute of Psychiatry, Psychology and Neuroscience, King's College London, London, United Kingdom; ^2^OASIS Service, South London and Maudsley NHS Foundation Trust, London, United Kingdom; ^3^Department of Brain and Behavioral Sciences, University of Pavia, Pavia, Italy; ^4^Department of Psychosis Studies, Institute of Psychiatry, Psychology and Neuroscience, King's College London, London, United Kingdom; ^5^COAST Service, South London and Maudsley NHS Foundation Trust, London, United Kingdom; ^6^LEO Early Intervention in Psychosis Service, South London and Maudsley NHS Foundation Trust, London, United Kingdom; ^7^Department of Social Genetics and Developmental Psychiatry, Institute of Psychiatry, Psychology and Neuroscience, King's College London, London, United Kingdom; ^8^Centre for Global Mental Health, Institute of Psychiatry, Psychology and Neuroscience, King's College London, London, United Kingdom; ^9^Centre for Implementation Science, Institute of Psychiatry, Psychology and Neuroscience, King's College London, London, United Kingdom; ^10^Department of Psychosis Studies, Institute of Psychiatry, Psychology and Neuroscience, King's College London, London, United Kingdom

**Keywords:** psychosis, schizophrenia, early intervention, SLaM, implementation, health service research

## Abstract

**Introduction:** Early Intervention for a first episode of Psychosis (EI) is essential to improve outcomes. There is limited research describing real-world implementation of EI services.

**Method:** Analysis of service characteristics, outcomes (described through a retrospective 2007–2017 Electronic Health Record (EHR) cohort study) and clinical research relating to the first 20 years of implementation of EI services in South London and Maudsley (SLaM) Trust.

**Results:** SLaM EI are standalone services serving 443,050 young individuals in South-London, where (2017) incidence of psychosis (58.3–71.9 cases per 100,000 person-years) is greater than the national average. From 2007–2017 (when the EHR was established), 1,200 individuals (62.67% male, mean age 24.38 years, 88.17% single; two-thirds of non-white ethnicity) received NICE-compliant EI care. Pathways to EI services came mainly (75.26%) through inpatient (39.83%) or community (19.33%) mental health services or Accident and Emergency departments (A&E) (16%). At 6 year follow-up 34.92% of patients were still being prescribed antipsychotics. The 3 month and 6 year cumulative proportions of those receiving clozapine were 0.75 and 7.33%; those compulsorily admitted to psychiatric hospitals 26.92 and 57.25%; those admitted to physical health hospitals 6.83 and 31.17%, respectively. Average 3 months and 6 year days spent in hospital were 0.82 and 1.85, respectively; mean 6 year attendance at A&E was 3.01. SLaM EI clinical research attracted £58 million grant income and numerous high-impact scientific publications.

**Conclusions:** SLaM EI services represent one of the largest, most established services of its kind, and are a leading model for development of similar services in the UK and worldwide.

## Introduction

Early Intervention for a first episode of psychosis (EI) is an established service model, with proven benefit in outcomes such as treatment discontinuation and hospitalization (1, 2). EI services have been operative since the late 1990s' in several parts of the world, and typically take care of individuals with a First-Episode Psychosis (FEP (2)). In England EI services have been established since the release of the IRIS guidelines in 1998 (3). In 1999 the UK Government required the provision of EI services across all of England, through the National Service Framework for Mental Health. In 2016, a new Access and Waiting Time Standard, released by National Health Service England set targets for time for assessments by EI teams (4). What characterizes EI services is their shared ethos of hope and recovery, and their assertive and holistic approach toward caring for young people with emerging mental disorder. Over the last 20 years or so, EI services in South London and Maudsley (SLaM) NHS Foundation Trust have played a pivotal role (5), as one of the first and largest EI services in the UK, integrated with a world-class research center (Institute of Psychiatry, Psychology, and Neuroscience, King's College London). Randomized controlled trials conducted in EI SLaM services demonstrated a reduced risk of psychotic relapses, hospital admission and disengagement from clinical services compared to standard care (5).

Given the limited implementation research describing EI service configuration, longer-term pragmatic outcomes and research impacts, the aim of this paper is to summarize the progresses made by SLaM EI service over the past two decades, focussing on the service characteristics, outcomes, and clinical research.

## Methods

### Design

Clinical, 6 year, retrospective, 2007–2017 Electronic Health Record (EHR) cohort study, to address outcomes, complemented by descriptive analyses to address service characteristics and research outputs of SLaM EI services, focusing on patients with a FEP. Although this study describes 20 years activity, cohort study data related to the last decade only because the local EHR was established only in 2007.

### Data Source

EHR data representing routine (also termed “real-world” to characterize cohort with high ecological validity) mental healthcare (6) from all patients managed by SLaM. SLaM was a pioneer of EHR, and the Trust is digitized and paper-free (7). SLaM is the major provider of secondary mental healthcare provision to its local catchment areas, and it is a legal requirement for SLaM healthcare professionals to keep EHR up-to-date (7). Whereas, many national registers capture only hospitalized patients, the SLaM EHR contains full clinical records of all patients in hospital and in community settings, continually updated throughout their care pathway, regardless of discharges from and/or referrals to other services. SLaM EHR has been employed in numerous publications (8–10); SLaM EHR-related methods and descriptive data are detailed elsewhere (6, 7, 11–14).

### Setting

SLaM is a National Health Service mental health Foundation Trust that provides secondary/specialist mental health care to a population of 1.36 million individuals (2018) in South London (Lambeth, Southwark, Lewisham, and Croydon boroughs). Broadly speaking, SLaM services are engineered in Clinical Academic Groups that represent real-world care pathways: acute care (for individuals with mental health crises), addictions, behavioral and developmental (which includes forensic services and services for neurodevelopmental disorders), child and adolescent, older adults and dementia, psychological medicine and integrated care (including general hospital liaison services, eating disorder services, perinatal psychiatry, neuropsychiatric services, and services for psychosexual conditions), and psychosis. The latter encompass EI services as well as recovery and rehabilitation services and services for individuals at Clinical High Risk of Psychosis (CHR-P) (15). The implementation of CHR-P services in SLaM has already been described in previous publications illustrating the characteristics of the Outreach And Support In South-London (OASIS) team in Lambeth and Southwark and in Lewisham and Croydon (16).

**Figure 1:**
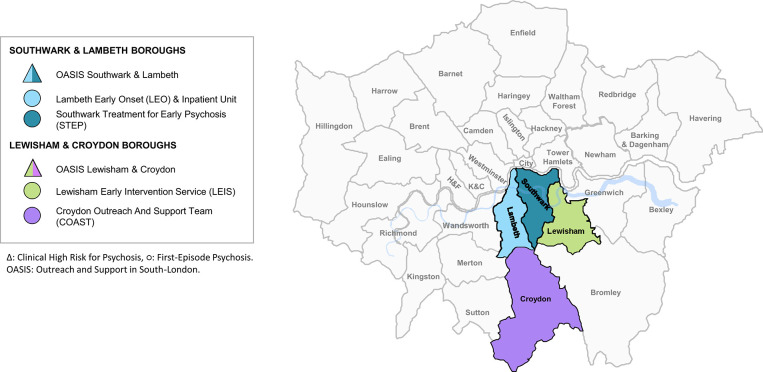
Early intervention services for a first episode of psychosis in South London and Maudsley (SLaM: Southwark, Lambeth, Lewisham, Croydon) and Clinical High Risk services. In triangles Clinical High Risk services, in squares first episode psychosis services. OASIS, Outreach and Support in South London prodromal service; STEP, Southwark Team for Early Psychosis; LEIS, Lewisham Early Intervention Service; LEO, Lambeth Early Onset Psychosis; COAST, Croydon Outreach Assessment Support Team. The inpatient unit for first-episode psychotic patients is not represented.

This study focused on the EI component of psychosis Clinical Academy Group, which includes FEP services only: (i) Lambeth Early Onset (LEO), (ii) Southwark Treatment for Early Psychosis (STEP), (iii) Lewisham Early Intervention Service (LEIS), (iv) Croydon Outreach and Assertive Support Team (COAST), and (v) a dedicated inpatient unit for individuals experiencing FEP psychosis, at Lambeth Hospital (LEO ward) (Figure 1). EI services were established in 2001 (17, 18). According to the National Institute of Clinical Excellence, EI services should provide care for individuals aged 14–35 (4), however, in the SLaM EHR, the typical entry age is of at least 18 years, with few cases of younger individuals aged 16 years. Furthermore, after April 2016 the upper age limit of EI services in the UK and SLaM was extended to 65 years (4). The current study pragmatically focuses on individuals aged 16–35 years to better capture the typical age range for EI services worldwide.

### Study Population

All individuals accessing SLaM from 2007 (when the EHR had been established) to 2017 (to allow a clinically meaningful follow-up, in keeping with other naturalistic follow up studies in similar populations (2)) and receiving any index ICD-10 diagnosis of non-organic mental disorder (defined in the eMethods 1 in Supplementary Material and formulated by the SLaM clinicians that were taking care of patients) were initially considered eligible, with no age restriction. The ICD-10 index diagnoses represented the first diagnosis received by patients upon when they were accepted by any SLaM service, thus representing their entry point into local healthcare. Individuals younger than 16 years or older than 35 years were then excluded. Of the remainder, those under care of local EI (i.e., FEP) services at any timepoint were included in follow-up. Therefore, this sample represent the typical patient group under the care of EI services in the UK. Approval for the study was granted by the Oxfordshire Research Ethics Committee C. Because the data set comprised de-identified data, informed consent was not required (7).

### Study Measures

All study measures were automatically extracted using the SLaM EHR (7).

#### Variables

Baseline descriptive variables included sociodemographic (age, gender, ethnicity, marital status), pathways to care (see below) and clinical characteristics (Health Of the Nation Outcome Scale [HONOS] (19) and primary ICD10 index diagnosis). The HoNOS is an NHS outcome measure that assesses general symptom severity and social functioning across time (19). It includes 12 items that measure behavior, impairment, symptoms and social functioning, each rated from 0 (no problem) to 4 (severe problem) (19) that are used to produce a total score.

#### Follow-Up

Follow-up started at the time of the recording of the baseline ICD-10 index diagnosis of mental disorder in the EHR, and was repeated at certain timepoints until 6 years (see below); it ended when the outcome of interest was recorded, or when follow-up had been completed. We additionally recorded duration of care under SLaM.

#### Outcome Measures

Our primary outcomes were point estimate and cumulative estimates (from intake to 3, 6, 12, 24, 48, and 72 months) of the proportion of FEP individuals first receiving: (i) treatment with antipsychotic medication; (ii) treatment with clozapine; (iii) compulsory admission to a mental health hospital (i.e., involving a Mental Health Act assessment); (iv) admission to physical health (general medical) hospitals; (v) numbers of days spent in any hospital for any reason; and (vi) number of contacts with Accident and Emergency (A&E) departments (by 72 months).

### Statistical Analysis

Firstly, we describe EI service characteristics: SLaM catchment area, service configuration, pathways to care and types of treatments offered. For the SLaM catchment area, we report the local incidence of psychosis (2017), estimated using PsyMaptic (http://www.psymaptic.org) (eMethods 2 in Supplementary Material). The EI service configuration was detailed by describing the staffing structure. Pathways to care are described by stratifying individuals under EI care across SLaM services that formulated their initial index diagnosis, the entry point into the healthcare system: EI services, Inpatient Mental Health Services (IMHS), Community Mental Health Services (CMHS), Accident and Emergency departments (A&E), Children and Adolescents Mental Health Services (CAMHS), Physical Health Services (PHS), and forensic mental health services. Types of treatment offered are described narratively.

We then report the results of the clinical register based EHR cohort study (2007–2017): baseline characteristics of EI patients and clinical outcomes. The EHR study was conducted according to the REporting of studies Conducted using Observational Routinely-collected health Data (RECORD) Statement (20). Baseline sociodemographic and clinical characteristics of the EHR sample (including missing data), are given with mean and SD for continuous variables, and absolute and relative frequencies for categorical variables. To estimate primary outcomes longitudinally, we describe two measures: point estimates and cumulative estimates of the proportion of individuals developing outcomes (i)–(iv) at different timepoints from 3 to 72 months. Outcomes (v) and (vi) were estimated longitudinally with descriptive analyses; box plots were used to describe the numbers of days spent in hospital for those admitted.

Lastly, we describe clinical research conducted within local EI services in the past 10 years, with particular focus on: national and international clinical research infrastructure, research capability, and clinical research outputs.

All analyses were conducted in STATA 14 (STATA Corp., TX, USA).

## Results

### Service Characteristics

#### Incidence of Psychosis Within SLaM

The general population aged 16–35 years in Lambeth was 115,751 in 2007 and 128,751 in 2017; in Southwark 105,232 in 2007 and 117,309 in 2017; in Lewisham 88,953 in 2007 and 96,378 in 2017; in Croydon 97,787 in 2007, and 100,612 in 2017 (21), total population of 407,722 in 2007 and 443,050 in 2017. Incidence of psychosis (in 2017) was: Lambeth 71.9 cases per 100,000 person-years, Southwark 69.6 cases per 100,000 person-years, Lewisham 71.3 cases per 100,000 person-years and Croydon 58.3 cases per 100,000 person-years, all substantially greater than the national average of 34.9 cases per 100,000 person-years.

#### Service Configuration

The current caseload and staffing configuration (to April 2020) of the four EI services in SLaM is given in eFigure 1 in Supplementary Material.

#### Pathways to Care

As shown in eFigure 2 in Supplementary Material, 117,983 individuals who accessed SLaM during the study period received primary index diagnosis of ICD-10 non-organic mental disorder were initially eligible. Of them, 73,174 individuals were excluded because they were outside the EI age range. Among the remaining 44,809 individuals aged 16–35, 1,200 were under care of EI services at any timepoint during the study period. The individuals excluded mostly represent patients who did not develop a FEP or who did so but outside the intake criteria of the local EI services and therefore do not represent the typical patient group which is cared by EI services.

**Figure 2:**
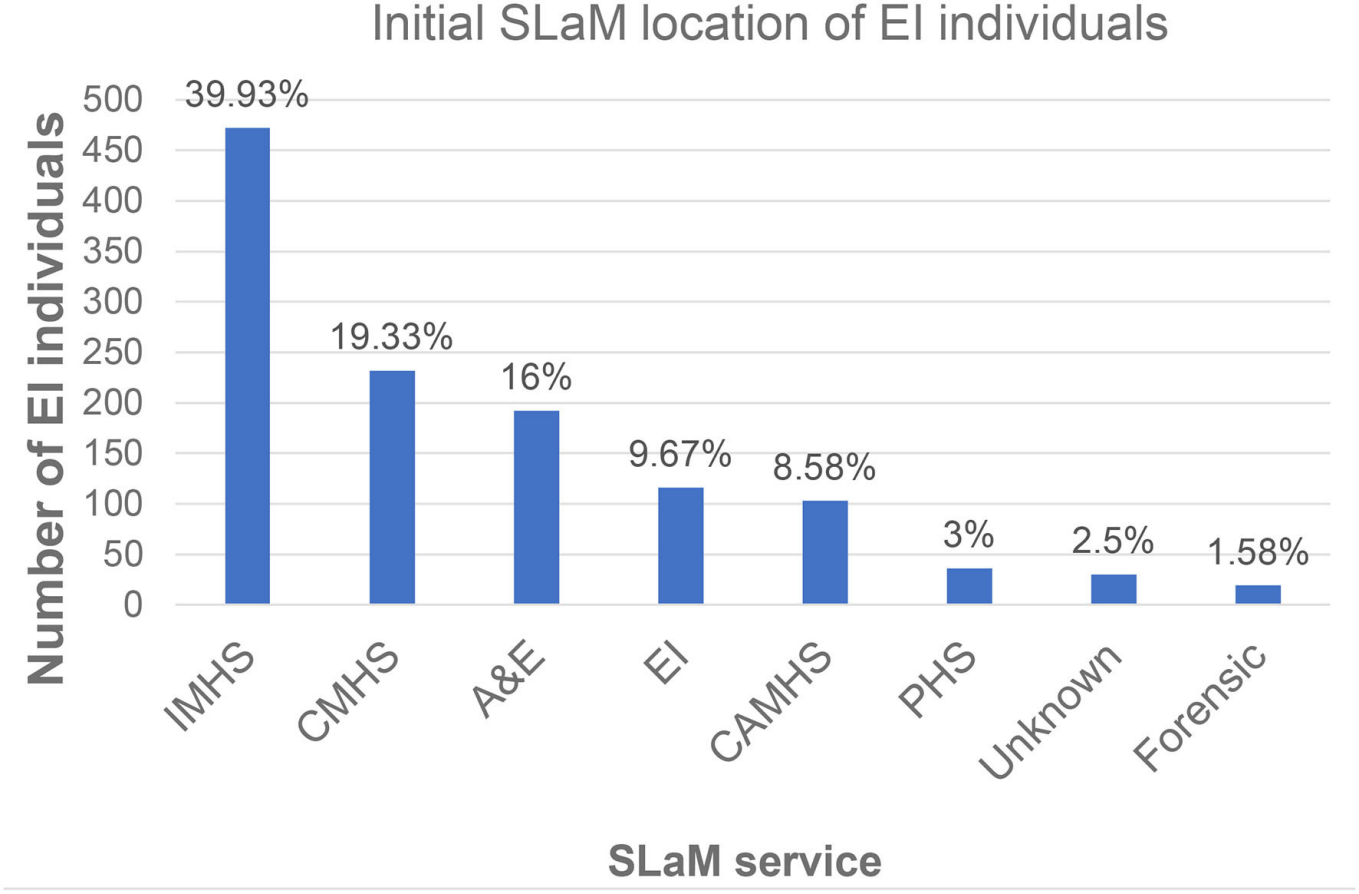
Pathways to care during a first episode of psychosis (*n* = 1200): initial location of EI individuals at the time of their index non-psychotic diagnosis (entry point into SLaM). IMHS, Inpatient Mental Health Services; CMHS, Community Mental Health Services; A&E, Accident and Emergency departments; CAMHS, Children and Adolescents Mental Health Services; PHS, Physical Health Services; EI, Early Intervention for psychosis. The blue bars indicate the count of EI individuals under each clinical service.

The location of EI individuals within SLaM services, at time of index diagnosis, is depicted in Figure 2. Most (75.26%) were under the care of IMHS (39.93%), CMHS (19.33%), or A&E (16%), followed by EI (9.67%), CAMHS (8.58%), PHS (3%), and forensic (1.58%).

#### Types of Treatment

SLaM EI services are multidisciplinary teams that offer assertive outreach intervention to patients experiencing FEP. STEP, LEIS, and COAST offer services for up to 3 years, while LEO provides services for up to 2 years. Services are provided with a transitional worker to ensure smooth transition from CAMHS to adult services. Services also use the Care Programme Approach framework, which allocated to each patient a specific Care Co-ordinator, and which ensures regular meetings focused on an integrated approach between social, housing, and health care services, and offer a comprehensive package of care encompassing:

- initial comprehensive assessments;
- medical reviews and pharmacological intervention, particularly antipsychotic medication. Patients and carers, whenever appropriate, are provided with information on medical treatment options, and supported to make informed decisions;
- early identification of treatment resistant psychoses;
- attention and the intervention strategies to dual diagnosis patients given the high co-morbidity with substance misuse;
- care co-ordination and regular contacts with patients and carers with the aim of building a therapeutic relationship, early relapse signs/relapse prevention, and making effective crisis plans;
- support with personal skills, social inclusion and social issues, such as benefits claims, housing and financial difficulties;
- psychological intervention;
- family therapy;
- physical health checks and intervention, as well as advice on healthy lifestyle;
- support to access education, employment related training, charity work, and paid employment, through vocational workers.

Services operate Monday–Friday from 09:00 to 17:00, and adhere to National Standards for Early Intervention in Psychosis Services (4).

### Outcomes (EHR Cohort Study)

#### Baseline Characteristics of EI Patients

As indicated in Table 1, 1,200 EI individuals (62.67% male) were under EI services during the study period. At presentation, mean age was 24.38 years (range 16–35); 88.17% were single; two-third were of non-white ethnicity (Black 54.00%, Asian 6.67%, Mixed 3.42%, Other 8%). Mean HoNOS at baseline was 12.93 (SD 12.05). Their primary ICD-10 index diagnosis was of non-psychotic mental disorders in about one third of the cases (Table 1).

**Table 1:** Baseline characteristics of the patients under the care of EI services for a first episode of psychosis.

		***N***	**Mean**	**SD**
**Age (years)**		1,200	24.38	5.32^a^
**HoNOS (total)**		1,182	12.93	12.05
		***N***	**Count**	**%**
**Gender**		1,200		
	Females		448	37.33
	Males		752	62.67
**Ethnicity**		1,200		
	White		335	27.92
	Asian		80	6.67
	Black		648	54.00
	Mixed		41	3.42
	Other		96	8.00
**Marital status**		1,200		
	Married		83	6.92
	Separated or divorced		39	3.25
	Single		1,058	88.17
	Other		20	1.66
**ICD-10 diagnostic spectrum**	F10-F19 Mental and behavioral disorders due to psychoactive substance use	1,200	119	9.92
	F20-F29 Schizophrenia, schizotypal and delusional disorders		833	69.42
	F30-F39 Mood [affective] disorders		164	13.67
	F40-F48 Neurotic, stress-related and somatoform disorders		51	4.25
	F50-F59 Behavioral syndromes associated with physiological disturbances and physical factors		9	0.75
	F60-F69 Disorders of adult personality and behavior		5	0.42
	F70-F79 Mental retardation		1	0.08
	F80-F89 Disorders of psychological development		5	0.042
	F90-F98 Behavioral and emotional disorders with onset usually occurring in childhood and adolescence		13	1.08

a *Range 16–35; n = 130 from 16 to 18. HoNOS, Health Of the Nation Outcome Scale*.

#### Clinical Outcomes

**Figure 3:**
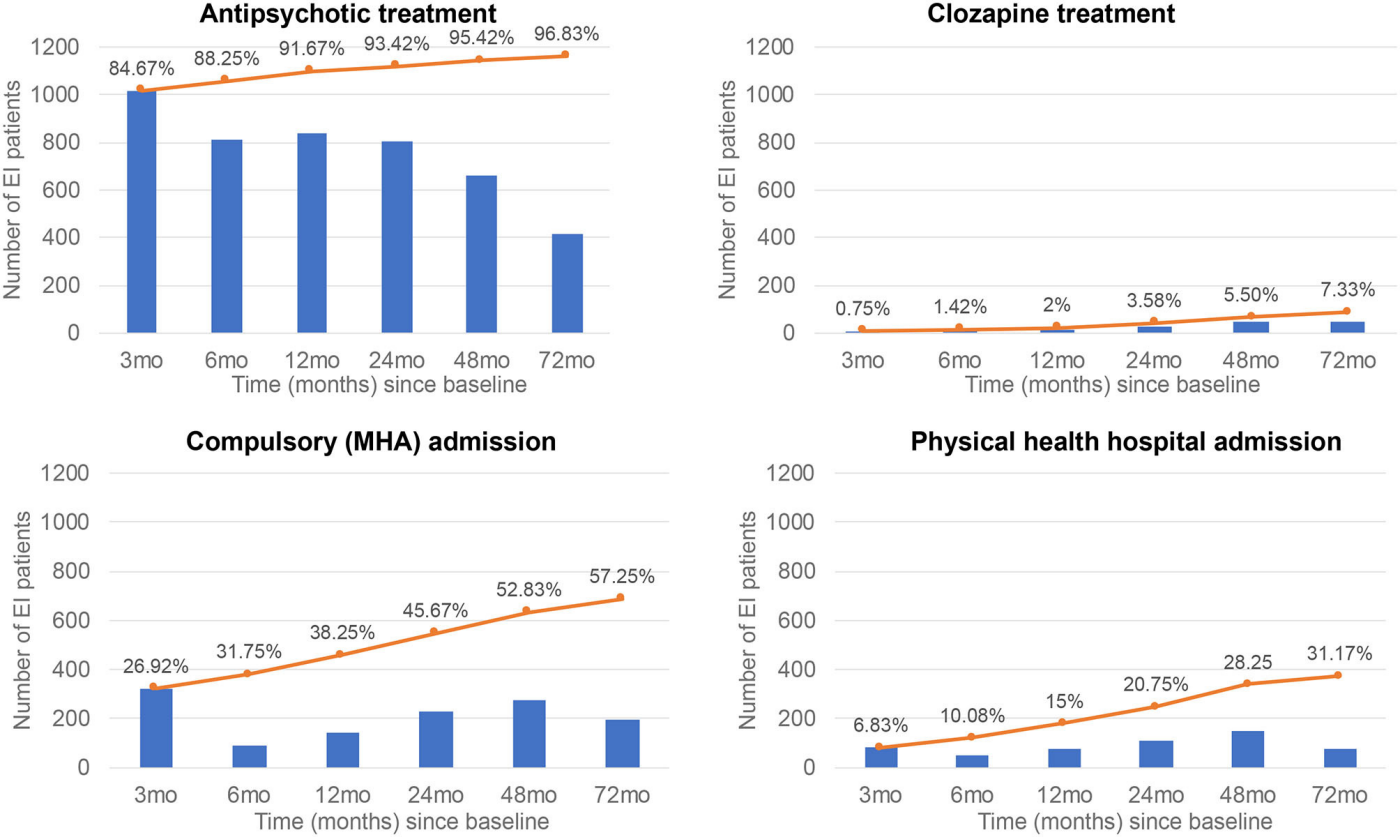
Real-world clinical outcomes in patients with a first-episode of psychosis (FEP) who were under EI care. Blue bars indicate time-point proportions, while orange lines indicate the cumulative proportions.

Duration of care lasted on average 1,341 days (SD 649.95). Among 1,200 EI individuals, 84.67% were prescribed antipsychotic treatment at 3 months, 67.83% at 6 months, 68.83% at 12 months, 66.83% at 24 months, 55.25% at 48 months, and 34.92% at 72 months. The cumulative proportion increased from 84.67% at 3 months to 96.83% at 72 months (Figure 3).

Among 1,200 EI individuals, 0.75% were prescribed clozapine at 3 months, 0.92% at 6 months, 1.00% at 12 months, 2.50% at 24 months, 3.92% at 48 months, and 4.17% at 72 months. The cumulative proportion increased from 0.75% at 3 months to 7.33% at 72 months (Figure 3).

Among 1,200 EI individuals, 26.92% were compulsorily admitted at 3 months, 7.42% at 6 months, 12.08% at 12 months, 18.83% at 24 months, 22.75% at 48 months, and 16.25% at 72 months. The cumulative proportion increased from 26.92% at 3 months to 57.25% at 72 months (Figure 3).

Among 1,200 EI individuals, 6.83% were admitted to physical health hospital at 3 months, 4.08% at 6 months, 6.50% at 12 months, 9.25% at 24 months, 12.75% at 48 months, and 6.42% at 72 months. The cumulative proportion increased from 6.83% at 3 months to 31.17% at 72 months (Figure 3).

Among 1,200 EI individuals, the average number of days spent in hospital was 0.82 days (SD = 6.75, range 0–92, median 0, 10–90th percentiles 0) at 3 months, 0.43 (SD = 4.85, range 0–92, median 0, 10–90th percentiles 0) at 6 months, 0.55 (SD = 7.08, range 0–182, median 0, 10–90th percentiles 0) at 12 months, 1.55 (SD = 13.63, range 0–292, median 0, 10–90th percentiles 0) at 24 months, 2.18 (SD = 22.63, range 0–494, median 0, 10–90th percentiles 0) at 48 months, 1.85 (SD = 24.43, range 0–681, median 0, 10–90th percentiles 0) at 72 months. Number of days spent in hospital among those who had been admitted is illustrated in eFigure 3 in Supplementary Material.

Among 1,200 EI individuals, the average number of contacts to A&E by 72 months was 3.01 (SD = 6.49, range 0–109).

### Clinical Research

EI services are closely linked to the Institute of Psychiatry, Psychology, and Neuroscience, being a component of the King's Health Partners Academic Health Sciences Center, with the aim of establishing a cutting-edge NHS service, where research innovation and clinical research are fully integrated.

#### National and International Clinical Research Infrastructures

SLaM EI services have specific links to the Department of Psychosis Studies at the Institute of Psychiatry, Psychology, and Neuroscience, which hosts the world's largest research group conducting research on psychosis, with over 1,300 scientific publications produced in the past 5 years. The Department of Psychosis Studies has received the maximum possible ranking (100% at 4^*^) for research environment in the 2014 UK Research Excellence Framework; its research impact was evaluated to be 100% “world-leading”.

Furthermore, at the National level, local EI services are integrated into cutting-edge clinical research through the National Institute of Health Research Mental Health Translational Research Collaboration Early Psychosis Workstream infrastructure, which includes major clinical research sites in the UK.

#### Clinical Research Capability

Leveraging the national and international infrastructures, over the past two decades, EI services in SLaM have played a strategic role leading or contributing to several global EI research consortia. Translational research within EI is conducted within the SLaM Biomedical Research Center, a major facility that supports local clinical infrastructure for hosting mental health research. Neurobiological research is mainly conducted at the Center for Neuroimaging Sciences, which allows a world-leading combination of application-oriented brain imaging (diffusion, functional, neurochemical, perfusion and structural imaging, and electrophysiology) analysis, as well as complementary research in imaging physics and analysis. Fluid (blood, plasma) and sample collection take place at the Maudsley BioResource for Mental Health. Finally, clinical research and follow-up are facilitated by existing EHR infrastructure (7). SLaM was awarded Global Digital Exemplar status by National Health Service England in 2017.

#### Clinical Research Output

Over the past 10 years, clinical research programmes led by EI services (detailed in eTable 1 in Supplementary Material) have been substantial, attracting about £58 million of grant income. While early studies demonstrated the benefits of EI services compared to standard care (5), more recently, research programmes have encompassed the study of social determinants and risk factors for FEP, reduction of delays to care and assessing treatment inequalities, and the development of digital technologies (smartphone apps, EHR screening, EHR linkage with smartphone apps) to measure FEP endophenotypes and outcomes. Furthermore, extensive neurobiological research has investigated normative brain charting and trajectories of brain changes during FEP. These studies have been enriched by neuroscientific approaches which have led to discovery and validation of potential biomarkers encompassing the neuromodulator dopamine, neurotransmitters such as glutamate and GABA, the N-methyl-D-aspartate receptor, and synaptic and neuronal autoantibodies in psychosis. A core component has been to combine neuroimaging and clinical prediction methods to advance stratified medicine, developing neurobiologically-based prediction models to forecast clinical outcomes such as relapses, response to treatments, treatment resistance or for the general optimisation of antipsychotic regimens during FEP. Finally, clinical research has strongly focused on experimental medicine and translational discoveries, with several research programmes testing efficacy of promising compounds (e.g., cannabidiol, potassium channel modulators), repurposing established drugs (e.g., clozapine), piloting innovative dietary interventions (e.g., vitamin D), or implementing physical health interventions (e.g., use of electronic cigarettes).

The scientific publications associated with these research programmes are numerous, and 10 illustrative examples are appended in eTable 2 in Supplementary Material. These examples encompass a variety of scientific approaches including randomized controlled trials, neuroscientific methods, observational cohort studies and risk prediction modeling.

## Discussion

To the best of our knowledge this is one of the few large-scale and long-term studies that has addressed the real-world implementation of EI services in the UK. The main strength of this study is to have provided a comprehensive empirical description of EI services around three core components: service characteristics, outcomes, and clinical research under routine clinical conditions.

With respect to service characteristics, SLaM EI teams serve an overall catchment area of 443,050 people aged 16–35 years, and are amongst the largest of their kinds in the UK and worldwide. Incidence of psychosis in SLaM (from 58.3 to 71.9 cases per 100,000 person-years (22)) is greater than the national average, and it is one of the highest worldwide (23). This may be accounted for by accumulation of several risk factors for psychosis such as immigration, higher numbers of people from ethnic minorities associated with psychosis (e.g., African/Afro-Caribbean, prevalence around 50%) and illicit substance misuse (24–26) (substance misuse data are not routinely available in the local EHR). In about one third of cases, the primary index diagnosis was of non-psychotic mental disorders.

The local population is also increasing substantially, resulting in increased needs such as housing, education, social care, and health care in general. All these increased demands will have reciprocal effects on absolute and relative inequalities in the population, a known determinant of health and mental health (27).

Local EI services accounted for some of these factors within a standalone model of care, which ensured sufficient resources and specialized staff for the management of early psychosis. Standalone models have been demonstrated to be more successful than other types of healthcare configurations in early psychosis (28, 29). The success of SLaM EI services is corroborated by the substantial number of individuals cared over the past 10 years, which represent one of the largest FEP cohorts worldwide.

Most individuals entered SLaM services through inpatient or community mental health teams, with subsequent referral to local EI services. Future outreach campaigns targeting these services, as well as the introduction of soft entry points such as the SLaM single point of access, may facilitate easier pathways into care, in line with advanced healthcare models established in Australia (30). A complementary approach may be to embed precision psychiatry in these refined healthcare systems (31), leveraging risk calculators that automatically screen EHR to detect individual risk of developing FEP (32, 33). These algorithms can also dynamically incorporate clinical information as it emerges (34, 35), for example access to A&E over follow-up (on average 3.01 over a 6 year period in EI patients, compared to an annualized median of 0.24 in the general population (36)). SLaM EI has recently piloted and implemented these tools in clinical routine practice (37).

With respect to outcomes, we were able to harness the potential of our EHR to better capture “real-world” outcomes in FEP, compared to other studies which have reported follow-up data on randomized controlled trial cohorts over a similar period of time (2). In line with current guidelines (38), the large majority of EI patients (84.67%) received the first-line recommendation- antipsychotic treatment- within 3 months, confirming the essential role of EI in reducing the duration of untreated psychosis (39). Interestingly, the proportion of those receiving antipsychotic medications declined over time, reaching 34.92% at 6 years. Interpreting these results in a naturalistic design is challenging; it may be possible that this low rate indexes good clinical outcomes in patients under the care of the local EI services. This is also consistent with the reduction of the point estimates of the proportion of EI patients undergoing compulsory admission to psychiatric hospital over time (from 26.92% at 3 months to 16.25% at 6 years; the cumulative proportions steadily increase over time). This change over time may also reflect side-effects of antipsychotics in a significant number of patients, as well as more acute presentations at first onset of illness (the first 5 years of illness being associated with greater variability in illness acuity (40)).

About one third-of (31.17%) EI patients had been admitted to physical health hospitals by end of the follow-up, presumably in light of increased identified physical co-morbidity. Over the years, SLaM EI services have implemented a comprehensive package of physical healthcare, which is expected to improve these outcomes, in keeping with recent recommendations (EI standards), and over time to contribute to the reduction of premature mortality of people with psychosis (41).

Around half of EI patients were compulsorily admitted to psychiatric hospital over a 6 year period, and this risk accumulated over time. Length of time spent in hospital increased over time. A relatively low proportion of EI patients received clozapine (7.33% at 6 years), and around 1% at 6 months/one year. In a cohort of 246 people with first onset schizophrenia spectrum disorders within SLaM, 23% had a treatment resistant illness (42), and in a similar sample of people with FEP, around 19% had treatment resistant illness (43). Therefore, even taking account of differences in being offered and taking clozapine, these numbers suggest an opportunity to improve access to clozapine in this cohort, in keeping with trial evidence in first episode schizophrenia (44). These findings (continued compulsory admission and low rates of clozapine use) represent opportunities for improvement within EI services, and are in keeping with general principles of EI and the recent EI standards (4).

With respect to clinical research we demonstrated that the synergic integration of National Health Services and academic infrastructure within an Academic Health Sciences Center has enabled delivery of cutting-edge output, advancing knowledge in the field of early psychosis, with £58 million of grant income and numerous scientific publications in high impact psychiatry journals. A number of large-scale stratified medicine, interventional and neurobiological studies are currently underway, with hope of further benefits for the many young people accessing local EI services.

The main limitations of the current study are inherited by its descriptive nature that prevented to test the impact of EI services compared to standard care. However, this type of analysis is not feasible using real-world EHR data that represent naturalistic pathways to care but would require controlled interventions and randomization that were outside the scope of the current investigation. Furthermore, these studies have already been conducted in SLaM (5). A recent meta-analysis comparing EI services vs. standard care for early psychosis has already demonstrated several benefits on different outcomes (2).

## Conclusions

With more than one thousand patients managed over two decades, SLaM EI represents one of the largest and most established services of its kind in the UK and worldwide. The cutting-edge quality of research evidence and the translational impact supports these services as a leading model for the development of similarly integrated services in the UK and worldwide.

## Data Availability Statement

The datasets presented in this article are not readily available because there was no ethical permission for data sharing. Requests to access the datasets should be directed to the corresponding author.

## Ethics Statement

The studies involving human participants were reviewed and approved by Oxfordshire Research Ethics Committee C. Written informed consent from the participants' legal guardian/next of kin was not required to participate in this study in accordance with the national legislation and the institutional requirements.

## Author Contributions

PF-P conceived the study and lead the data analysis together with SJ. SL, MF, EI, GT, and PM provided substantial input to the interpretation of the results. All authors drafted the manuscript. All authors contributed to the article and approved the submitted version.

## Conflict of Interest

GT was supported by the National Institute for Health Research (NIHR) Applied Research Collaboration South London at King's College London NHS Foundation Trust, and by the NIHR Asset Global Health Unit award. GT also received support from the National Institute of Mental Health of the National Institutes of Health under award number R01MH100470 (Cobalt study). GT was supported by the UK Medical Research Council in relation to the Emilia (MR/S001255/1) and Indigo Partnership (MR/R023697/1) awards. PF-P had received research fees or honoraria from Lundbeck, Angelini, Menarini, and Boehringer Ingelheim. SJ has received honoraria from Snovian for educational talks, and King's College London has received fees for educational talks SJ has given for Lundbeck. The remaining authors declare that the research was conducted in the absence of any commercial or financial relationships that could be construed as a potential conflict of interest.
