# Supplementary material for: Early Intervention Services for First Episode of Psychosis in South London and the Maudsley (SLaM): 20 Years of Care and Research for Young People
Source: Front Psychiatry. 2020 Nov 24;11:577110. doi: 10.3389/fpsyt.2020.577110 (PMC7732476; doi:10.3389/fpsyt.2020.577110)
Supplement: Supplementary file 1 [file Data_Sheet_1.docx]

**eMethods 1**. ICD-10 index diagnoses considered at baseline

(F10-F19 Mental and behavioural disorders due to psychoactive substance use; F20-F29 Schizophrenia, schizotypal and delusional disorders; F30-F39 Mood [affective] disorders; F40-F48 Neurotic, stress-related and somatoform disorders; F50-F59 Behavioural syndromes associated with physiological disturbances and physical factors; F60-F69 Disorders of adult personality and behaviour; F70-F79 Mental retardation; F80-F89 Disorders of psychological development; F90-F98 Behavioural and emotional disorders with onset usually occurring in childhood and adolescence)

**eMethods 2**

To estimate the predicted number of FEP cases in the local sociodemographically-matched general population, we used PsyMaptic (<http://www.psymaptic.org>). PsyMaptic employs core sociodemographic predictors that characterise the local general population to produce local incidence of psychosis for the age range 16-35. Details on PsyMaptic have been given elsewhere[1]. Because incidence of psychosis in England remained stable over the past decades[2], we assumed a constant incidence of psychosis from 2007 to 2017.

**eFigure 1**. Staffing structure of EI services (up to April 2020) across South London And Maudsley (SLaM) NHS Foundation Trust: Croydon Outreach Assessment Support Team (COAST), Lewisham Early Intervention Service (LEIS), Southwark Team for Early Psychosis (STEP), Lambeth Early Onset Psychosis (LEO). The average yearly caseload for each EI service is represented on the left side of the figure. The numbers indicate the relative full-time equivalent of each professional figure.

**eFigure 2.** Flow chart of study population

Patients receiving a first index diagnosis of any ICD-defined mental disorder within SLaM in the period 2007-2017

(n=117983)

Patients younger than 16 years or older than 35 years (n=73174)

Patients eligible for EI care (16<=aged>=35 years)

(n=44809)

Patients not under the EI care (n=43609)

Patients under the care of EI services at any timepoint during 2007-2017

(n=1200)

**eFigure 3**. Number of days spent in hospital among EI individuals who had been admitted to hospital.


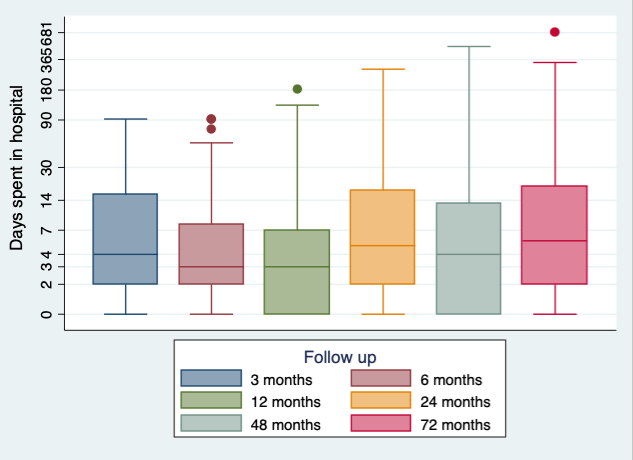


| **eTable 1 EI (FEP) research programme** | **Funder** | **Amount** | **Period** |
| --- | --- | --- | --- |
| Developing Mobile Digital Technologies to Measure Stress-Biomarker Signatures Across Psychotic Illness Stages | BBRF | GPB 50000 | 2020-2022 |
| Repurposing Clozapine in psychosis | Maudsley Charity | GBP 458000 | 2020-2022 |
| Normative brain charting for predicting and stratifying psychosis | WT | GPB 494930 | 2019-2021 |
| Using smartphone-based personal sensing to understand and predict risk of psychotic relapse at the individual level | MRC | GBP 754342 | 2019-2023 |
| PSYSCAN: Translating neuroimaging findings from research into clinical practice | EC | Eur 6000000 | 2014-2020 |
| STEP: Stratification & Treatment in Early Psychosis | WT | GBP 15000000 | 2020-2025 |
| King’s Mental Health Data Pathfinder award | MRC | GBP 1479000 | 2018-2020 |
| Linking electronic health records with passive smartphone activity data to predict outcomes in psychotic disorders | MRC | GBP 326858 | 2018-2021 |
| Measuring dopamine synthesis capacity in people with psychotic depression, and comparison to other psychotic illnesses | RCP (Edin.) | GBP 156046 | 2018-2021 |
| The role of glutamate and N-methyl-D-aspartate receptor (NMDAR) in schizophrenia | RCP | GBP 223955 | 2017-2019 |
| NIHR Biomedical Research Centre. Funding for Financial Years 2017/18, 2018/19 | NIHR | GBP 20000000 | 2017-2022 |
| Stress and GABA in the pathogenesis of psychosis | WT | GBP 1089387 | 2017-2022 |
| Neurobiology of response to clozapine in treatment resistant schizophrenia | MRC | GBP 68000 | 2017-2018 |
| Using smartphone technologies to investigate the effects of the physical and interpersonal environment on coping strategies in early psychosis | BBRF | GBP 44324 | 2017-2020 |
| Biological markers of stress and inflammation across clinical stages of schizophrenia: from early at risk states to chronic illness | WT | GBP 250000 | 2015-2019 |
| Does Kv3 channel modulation reduce dopamine synthesis capacity and reduce abnormal neural response in schizophrenia? | Innovate UK | GBP 364577 | 2015-2018 |
| Synaptic and neuronal autoantibodies in psychosis (SNAP): clinical significance, neuroimaging correlates, infective/immune risk factors | WT | GBP 272696 | 2015-2019 |
| STRATA: Schizophrenia: Treatment Resistance and Therapeutic Advances | MRC | GBP 4012041 | 2014-2019 |
| D-fend – Vitamin D first episode neuroprotection design | SMRI | GBP 861204 | 2014-2020 |
| Neurobiology of response to clozapine in treatment resistant schizophrenia | MRC | GBP 637868 | 2014-2017 |
| Acceptability, patterns of use and safety of electronic cigarette in people with mental illness: a pilot study (APUS-eCig) | Maudsley Charity | GBP 153003 | 2014-2017 |
| Social context and the development, persistence and outcome of psychotic symptoms in the general population | WT | GBP 224765 | 2013-2017 |
| Is there a change in dopaminergic function with the onset of psychosis? | BBRF | GBP 25986 | 2013-2015 |
| Evaluation of cannabidiol as a treatment for the early phase of psychosis | WT | GBP 99221 | 2013-2014 |
| Glutamate and treatment response in psychosis | BBRF | GBP 33568 | 2013-2016 |
| Predicting clinical and functional outcomes in psychosis using machine learning | MRC | GBP 174823 | 2013-2016 |
| Predicting drug response in schizophrenia: are clinical & cognitive tests a cost-effective alternative to neuroimaging? | MRC | GBP 205228 | 2013-2014 |
| Trajectory of Brain Structure and Function before and after the Onset of Psychosis: a Longitudinal Multicentre Study | MRC | GBP 1227953 | 2012-2018 |
| Neurobiological determinants of treatment response in psychosis | WT | GBP 327434 | 2012-2017 |
| Prediction of risk of relapse of psychosis -examining the interaction between genes and substance use (PREDICT) | NIHR | GBP 817060 | 2012-2017 |
| Reducing delays in treating resistant schizophrenia and assessing treatment inequalities | Maudsley Charity | GBP 297817 | 2012-2019 |
| Neurobiological factors underlying the onset of psychosis | WT | GBP 1,054,325 | 2011-2017 |
| Structural disorganisation in psychosis and its functional consequences | WT | GBP 180339 | 2011-2014 |
| Optimisation of Treatment and Management of Schizophrenia in Europe | EC | GBP 1455216 | 2010-2016 |
| Relationship between Dopamine & Glutamate Dysfunction in Schizophrenia | BBRF | GBP 63666 | 2010-2013 |
| BBRF: Brain and Behavior Research Foundation; MRC, Medical Research Council; WT, Wellcome Trust; NIHR: National Institute of Health Research; NIMH: National Institute of Mental Health; EC: European Commission; G&T: Guy's and St Thomas' Charity; RCP (Edin.) Royal College of Physicians, Edinburgh; RCPsych: Royal College of Psychiatrists; SMRI: Stanley Medical Research Institute. | | | |

| **eTable 2** Ten EI-related top scientific publication (published in JAMA Psychiatry or Lancet Psychiatry) over the past 10 years | | |
| --- | --- | --- |
| **Author** | **Type** | **Main finding** |
| [3] | [^18^F]-MPEP-d2 + [^11^C]-MePPEP PET | Availability of CB1R was lower in antipsychotic-treated and untreated cohorts relative to matched controls |
| [4] | [^18^F]-DOPA  PET + MRS | Cortical glutamate dysfunction related to subcortical dopamine synthesis capacity in psychosis, though not in controls |
| [5] | RCT | Cannabidiol has beneficial effects in patients with established schizophrenia |
| [6] | [^18^F]-DOPA  PET | Dopamine synthesis capacity is positively correlated with positive psychotic symptom severity across both bipolar disorders and schizophrenia |
| [7] | Prediction modelling | Development and external validation of a clinically-based, transdiagnostic risk prediction model for the automatic detection of individuals with emerging psychosis |
| [8] | Observational | Adverse effects associated with use of cannabis after the onset of a FEP depend on patterns of use |
| [9] | Observational | Availability of high potency cannabis in South London may explain 24% of FEP |
| [10] | MRI | Gyrification appears to be a useful predictor of antipsychotic treatment response |
| [11] | [^18^F]-DOPA  PET | Antipsychotic response in FEP is related to baseline dopamine synthesis capacity |
| [12] | [^18^F]-DOPA  PET | Antipsychotic treatment has no effect on dopamine synthesis capacity in FEP |
| [10] | MRI | Gyrification appears to be a useful predictor of antipsychotic treatment response |

RCT: Randomised Controlled Trial; [18F]-DOPA PET: Fluorine-18-L-Dihydroxyphenylalanine Positron Emission Tomography; MRS: Magnetic Resonance Spectroscopy;  [18F]-MPEP-d2: fluoride 18-labeled FMPEP-d2; [11C]-MePPEP: carbon 11-labeled MePPEP; MRI: Magnetic Resonance Imaging.

Bibliografia

[1] J.B. Kirkbride, D. Jackson, J. Perez, D. Fowler, F. Winton, J.W. Coid, R.M. Murray, and P.B. Jones, A population-level prediction tool for the incidence of first-episode psychosis: translational epidemiology based on cross-sectional data. BMJ Open 3 (2013).

[2] J.B. Kirkbride, A. Errazuriz, T.J. Croudace, C. Morgan, D. Jackson, J. Boydell, R.M. Murray, and P.B. Jones, Incidence of Schizophrenia and Other Psychoses in England, 1950–2009: A Systematic Review and Meta-Analyses. PloS one 7 (2012) e31660.

[3] F. Borgan, H. Laurikainen, M. Veronese, T.R. Marques, M. Haaparanta-Solin, O. Solin, T. Dahoun, M. Rogdaki, R.K. Salokangas, M. Karukivi, M. Di Forti, F. Turkheimer, J. Hietala, O. Howes, and M. Group, In Vivo Availability of Cannabinoid 1 Receptor Levels in Patients With First-Episode Psychosis. JAMA Psychiatry (2019).

[4] S. Jauhar, R. McCutcheon, F. Borgan, M. Veronese, M. Nour, F. Pepper, M. Rogdaki, J. Stone, A. Egerton, F. Turkheimer, P. McGuire, and O.D. Howes, The relationship between cortical glutamate and striatal dopamine in first-episode psychosis: a cross-sectional multimodal PET and magnetic resonance spectroscopy imaging study. Lancet Psychiatry 5 (2018) 816-823.

[5] P. McGuire, P. Robson, W.J. Cubala, D. Vasile, P.D. Morrison, R. Barron, A. Taylor, and S. Wright, Cannabidiol (CBD) as an Adjunctive Therapy in Schizophrenia: A Multicenter Randomized Controlled Trial. Am J Psychiatry 175 (2018) 225-231.

[6] S. Jauhar, M.M. Nour, M. Veronese, M. Rogdaki, I. Bonoldi, M. Azis, F. Turkheimer, P. McGuire, A.H. Young, and O.D. Howes, A Test of the Transdiagnostic Dopamine Hypothesis of Psychosis Using Positron Emission Tomographic Imaging in Bipolar Affective Disorder and Schizophrenia. JAMA Psychiatry 74 (2017) 1206-1213.

[7] P. Fusar-Poli, G. Rutigliano, D. Stahl, C. Davies, I. Bonoldi, T. Reilly, and P. McGuire, Development and validation of a clinically based risk calculator for the transdiagnostic prediction of psychosis. Jama Psychiatry 74 (2017) 493-500.

[8] T. Schoeler, N. Petros, M. Di Forti, E. Klamerus, E. Foglia, O. Ajnakina, C. Gayer-Anderson, M. Colizzi, D. Quattrone, I. Behlke, S. Shetty, P. McGuire, A.S. David, R. Murray, and S. Bhattacharyya, Effects of continuation, frequency, and type of cannabis use on relapse in the first 2 years after onset of psychosis: an observational study. Lancet Psychiatry 3 (2016) 947-953.

[9] M. Di Forti, A. Marconi, E. Carra, S. Fraietta, A. Trotta, M. Bonomo, F. Bianconi, P. Gardner-Sood, J. O'Connor, M. Russo, S.A. Stilo, T.R. Marques, V. Mondelli, P. Dazzan, C. Pariante, A.S. David, F. Gaughran, Z. Atakan, C. Iyegbe, J. Powell, C. Morgan, M. Lynskey, and R.M. Murray, Proportion of patients in south London with first-episode psychosis attributable to use of high potency cannabis: a case-control study. Lancet Psychiatry 2 (2015) 233-8.

[10] L. Palaniyappan, T.R. Marques, H. Taylor, R. Handley, V. Mondelli, S. Bonaccorso, A. Giordano, G. McQueen, M. DiForti, A. Simmons, A.S. David, C.M. Pariante, R.M. Murray, and P. Dazzan, Cortical folding defects as markers of poor treatment response in first-episode psychosis. JAMA Psychiatry 70 (2013) 1031-40.

[11] S. Jauhar, M. Veronese, M.M. Nour, M. Rogdaki, P. Hathway, F.E. Turkheimer, J. Stone, A. Egerton, P. McGuire, S. Kapur, and O.D. Howes, Determinants of treatment response in first-episode psychosis: an (18)F-DOPA PET study. Mol Psychiatry 24 (2019) 1502-1512.

[12] S. Jauhar, M. Veronese, M.M. Nour, M. Rogdaki, P. Hathway, S. Natesan, F. Turkheimer, J. Stone, A. Egerton, P. McGuire, S. Kapur, and O.D. Howes, The Effects of Antipsychotic Treatment on Presynaptic Dopamine Synthesis Capacity in First-Episode Psychosis: A Positron Emission Tomography Study. Biol Psychiatry 85 (2019) 79-87.

Bibliografia

[1] J.B. Kirkbride, A. Errazuriz, T.J. Croudace, C. Morgan, D. Jackson, J. Boydell, R.M. Murray, and P.B. Jones, Incidence of Schizophrenia and Other Psychoses in England, 1950–2009: A Systematic Review and Meta-Analyses. PloS one 7 (2012) e31660.

[2] F. Borgan, H. Laurikainen, M. Veronese, T.R. Marques, M. Haaparanta-Solin, O. Solin, T. Dahoun, M. Rogdaki, R.K. Salokangas, M. Karukivi, M. Di Forti, F. Turkheimer, J. Hietala, O. Howes, and M. Group, In Vivo Availability of Cannabinoid 1 Receptor Levels in Patients With First-Episode Psychosis. JAMA Psychiatry (2019).

[3] S. Jauhar, R. McCutcheon, F. Borgan, M. Veronese, M. Nour, F. Pepper, M. Rogdaki, J. Stone, A. Egerton, F. Turkheimer, P. McGuire, and O.D. Howes, The relationship between cortical glutamate and striatal dopamine in first-episode psychosis: a cross-sectional multimodal PET and magnetic resonance spectroscopy imaging study. Lancet Psychiatry 5 (2018) 816-823.

[4] P. McGuire, P. Robson, W.J. Cubala, D. Vasile, P.D. Morrison, R. Barron, A. Taylor, and S. Wright, Cannabidiol (CBD) as an Adjunctive Therapy in Schizophrenia: A Multicenter Randomized Controlled Trial. Am J Psychiatry 175 (2018) 225-231.

[5] S. Jauhar, M.M. Nour, M. Veronese, M. Rogdaki, I. Bonoldi, M. Azis, F. Turkheimer, P. McGuire, A.H. Young, and O.D. Howes, A Test of the Transdiagnostic Dopamine Hypothesis of Psychosis Using Positron Emission Tomographic Imaging in Bipolar Affective Disorder and Schizophrenia. JAMA Psychiatry 74 (2017) 1206-1213.

[6] P. Fusar-Poli, G. Rutigliano, D. Stahl, C. Davies, I. Bonoldi, T. Reilly, and P. McGuire, Development and validation of a clinically based risk calculator for the transdiagnostic prediction of psychosis. Jama Psychiatry 74 (2017) 493-500.

[7] T. Schoeler, N. Petros, M. Di Forti, E. Klamerus, E. Foglia, O. Ajnakina, C. Gayer-Anderson, M. Colizzi, D. Quattrone, I. Behlke, S. Shetty, P. McGuire, A.S. David, R. Murray, and S. Bhattacharyya, Effects of continuation, frequency, and type of cannabis use on relapse in the first 2 years after onset of psychosis: an observational study. Lancet Psychiatry 3 (2016) 947-953.

[8] M. Di Forti, A. Marconi, E. Carra, S. Fraietta, A. Trotta, M. Bonomo, F. Bianconi, P. Gardner-Sood, J. O'Connor, M. Russo, S.A. Stilo, T.R. Marques, V. Mondelli, P. Dazzan, C. Pariante, A.S. David, F. Gaughran, Z. Atakan, C. Iyegbe, J. Powell, C. Morgan, M. Lynskey, and R.M. Murray, Proportion of patients in south London with first-episode psychosis attributable to use of high potency cannabis: a case-control study. Lancet Psychiatry 2 (2015) 233-8.

[9] L. Palaniyappan, T.R. Marques, H. Taylor, R. Handley, V. Mondelli, S. Bonaccorso, A. Giordano, G. McQueen, M. DiForti, A. Simmons, A.S. David, C.M. Pariante, R.M. Murray, and P. Dazzan, Cortical folding defects as markers of poor treatment response in first-episode psychosis. JAMA Psychiatry 70 (2013) 1031-40.

[10] S. Jauhar, M. Veronese, M.M. Nour, M. Rogdaki, P. Hathway, F.E. Turkheimer, J. Stone, A. Egerton, P. McGuire, S. Kapur, and O.D. Howes, Determinants of treatment response in first-episode psychosis: an (18)F-DOPA PET study. Mol Psychiatry 24 (2019) 1502-1512.

[11] S. Jauhar, M. Veronese, M.M. Nour, M. Rogdaki, P. Hathway, S. Natesan, F. Turkheimer, J. Stone, A. Egerton, P. McGuire, S. Kapur, and O.D. Howes, The Effects of Antipsychotic Treatment on Presynaptic Dopamine Synthesis Capacity in First-Episode Psychosis: A Positron Emission Tomography Study. Biol Psychiatry 85 (2019) 79-87.
